# Fingolimod therapy is not effective in a mouse model of spontaneous autoimmune peripheral polyneuropathy

**DOI:** 10.1038/s41598-018-23949-4

**Published:** 2018-04-04

**Authors:** Petra Huehnchen, Wolfgang Boehmerle, Matthias Endres

**Affiliations:** 1 Charité – Universitätsmedizin Berlin, corporate member of Freie Universität Berlin, Humboldt-Universität zu Berlin, and Berlin Institute of Health, Klinik und Hochschulambulanz für Neurologie, Berlin, Germany; 2 Charité – Universitätsmedizin Berlin, corporate member of Freie Universität Berlin, Humboldt-Universität zu Berlin, and Berlin Institute of Health, Cluster of Excellence NeuroCure, Berlin, Germany; 3 Berlin Institute of Health, 10178 Berlin, Germany; 4 Charité – Universitätsmedizin Berlin, corporate member of Freie Universität Berlin, Humboldt-Universität zu Berlin, and Berlin Institute of Health, Center for Stroke Research Berlin, Berlin, Germany; 5 German Center for Neurodegenerative Diseases (DZNE), Berlin, Germany; 6 DZHK (German Center for Cardiovascular Research), partner site Berlin, Berlin, Germany

## Abstract

Chronic inflammatory demyelinating polyradiculoneuropathy (CIDP) is an autoimmune disorder, which causes progressive sensory and motor deficits and often results in severe disability. Knockout of the co-stimulatory protein CD86 in mice of the non-obese diabetic background (NoD.129S4-Cd86^*tm1Shr*^/JbsJ) results in the development of a spontaneous autoimmune peripheral polyneuropathy (SAPP). We used this previously described transgenic model to study the effects of the sphingosine-1-phosphate receptor agonist fingolimod on SAPP symptoms, functional and electrophysiological characteristics. Compared to two control strains, knockout of CD86 in NOD mice (CD86^−/−^ NOD) resulted in progressive paralysis with distinct locomotor deficits due to a severe sensory-motor axonal-demyelinating polyneuropathy as assessed by electrophysiological measurements. We started fingolimod treatment when CD86^−/−^ NOD mice showed signs of unilateral hind limb weakness and continued at a dose of 1 mg/kg/day for eight weeks. We did not observe any beneficial effects of fingolimod regarding disease progression. In addition, fingolimod did not influence the functional outcome of CD86^−/−^ NOD mice compared to vehicle treatment nor any of the electrophysiological characteristics. In summary, we show that fingolimod treatment has no beneficial effects in autoimmune polyneuropathy, which is in line with recent clinical data obtained in CIDP patients.

## Introduction

Chronic inflammatory demyelinating polyradiculoneuropathy (CIDP) is the most common treatable autoimmune neuropathy^1^. It is typically characterized by progressive proximal and distal paresis as well as sensory deficits, which correspond to severe demyelination and secondary axonal loss of peripheral nerves (reviewed by^2^). However, many phenotypic subtypes of this condition have been described including, but not limited to, sensory predominant CIDP, motor dominant CIDP, multifocal acquired demyelinating sensory and motor neuropathy (MADSAM), distal acquired demyelinating symmetric neuropathy (DADS) or focal CIDP (reviewed by^3^). The variety of clinical presentation often complicates and delays clinical diagnosis and treatment. The commonly accepted theory to date is that both cell-mediated and humoral mechanisms contribute to the pathogenesis of CIDP^1,4–6^. The treatment with plasma exchange or intravenous immunoglobulins (IVIg) is typically very effective in CIDP patients and suggests the involvement of pathogenic autoantibodies^7^. The role of cellular immune mechanisms in CIDP pathogenesis on the other hand is substantiated by the presence of inflammatory infiltrates in nerve biopsies, an altered expression of inflammatory mediators such as cytokines and the contribution of pathogenic T cells to the development of a CIDP-like phenotype in animal studies^8,9^. It has been shown in CIDP that CD4^+^ T cells secrete pro-inflammatory cytokines including Interleukin (IL)-2, interferon-γ (IFN-γ) and IL-17^10^, which activate macrophages and upregulate adhesion molecules such as vascular cell adhesion molecule (VCAM)-1, endothelial leukocyte adhesion molecule (ELAM)-1 and intercellular adhesion molecule (ICAM)-1 in the blood vessels of peripheral nerves^11^. Activated T cells can thus bind to the adhesion molecules and cross the blood-nerve-barrier and contribute to its breakdown. Amongst those highly pathogenic T cells are T_H_17 cells, which were found to be elevated in serum and cerebrospinal fluid samples of patients with active CIDP^12^. Corresponding to this finding, it was shown that T_H_17 cells are the determining factor of disease severity, but not target specificity, in a transgenic ICAM-1 deficient mouse model of CIDP^13^.

Currently, treatment of CIDP involves corticosteroids, IVIg and plasmapheresis as first line therapies (reviewed by^14^). In addition, beneficial effects of the CD20-antibody rituximab have been demonstrated^15^. However, some CIDP patients do not respond to established treatments or develop severe side effects. Therefore, new treatment options are required. The sphingosine-1-phosphate analogue fingolimod has become a crucial part of the immunomodulatory treatment of multiple sclerosis. It works by binding to the sphingosine-1-phosphate (S1P-)-receptor, which in turn becomes internalized and lymphocytes remain sequestered in lymph nodes, preventing them to infiltrate the nervous system. Fingolimod has been proven to be beneficial in an animal model of Guillain-Barré-Syndrome^16^ and a phase II/III clinical trial in CIDP patients has been underway (ClinicalTrials identifier NCT01625182)^17^. In this study, we were interested to see whether fingolimod treatment could prevent progression of autoimmune neuropathy as assessed by functional and electrophysiological tests.

## Results

We used a previously described transgenic mouse model, which mimics clinical, electrophysiological and histological characteristics of CIDP. In this model, knockout of the costimulatory molecule CD86 (CD86^−/−^) in non-obese diabetic (NOD) mice (NoD.129S4-Cd86^*tm1Shr*^/JbsJ) results in the spontaneous development of an autoimmune peripheral polyneuropathy (SAPP) starting at approx. 20 weeks of age with female mice showing a much higher incidence than males^18^.

### Development of progressive paralysis and severe demyelination of peripheral nerves in CD86^−/−^ NOD mice

**Figure 1:**
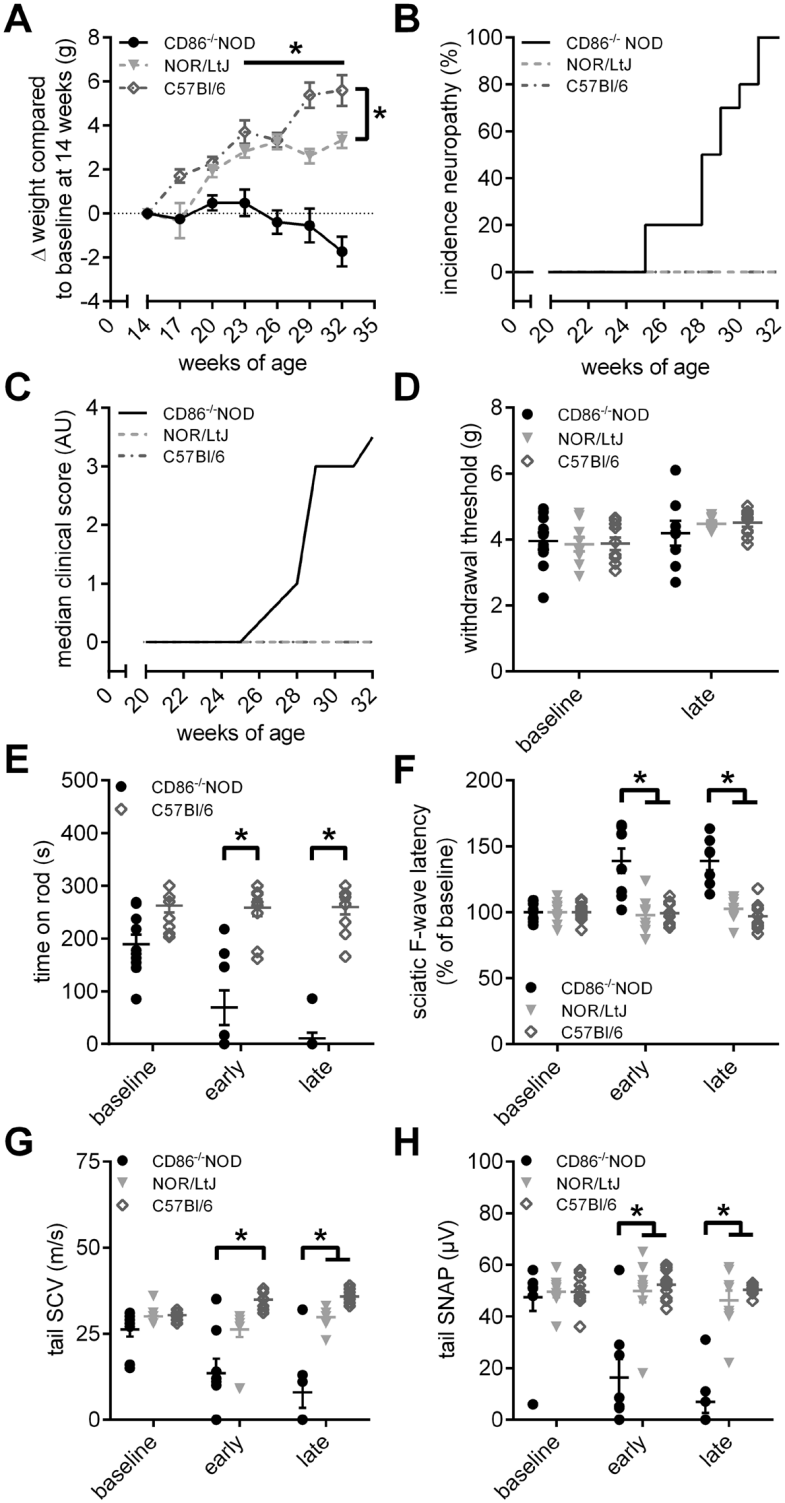
Clinical, behavioral and electrophysiological phenotype of CD86^−/−^ NOD transgenic mice. (**A**) CD86^−/−^ NOD mice showed a slower weight gain compared to C57Bl/6 and NOR/LtJ control mice and started losing weight with onset of SAPP symptoms, which was significant from weeks 23 of age to the end of the experiment. NOR/LtJ control mice also showed a slower weight gain beginning week 29 of age compared to C57Bl/6 control mice. (**B**) CD86^−/−^ NOD mice developed clinical signs of neuropathy (paralysis) as early as 25 weeks of age. None of the control mice showed signs of neuropathy. (**C**) Paralysis steadily progressed in CD86^−/−^ NOD mice until all mice had to be sacrificed according to humane endpoints by 32 weeks of age when reaching a clinical score of >3.5. (**D**) We did not observe any signs of mechanical hypersensitivity in any of the mice, while (**E**) locomotor function significantly decre-ased in CD86^−/−^ NOD mice over time. (**F**) In parallel, CD86^−/−^ NOD mice developed a significant increase of the F-wave latency of the sciatic nerve. (**G**) The sensory nerve conduction velocity (SCV) as well as (**H**) the sensory nerve action potential amplitude (SNAP) of the tail nerve decreased in CD86^−/−^ NOD mice. Statistical analysis: (**A**,**D**,**F**) 2-way ANOVA with Sidak post hoc, (**B**) Kaplan-Meier analysis with Mantel-Cox test, (**E**,**G**,**H**) Kruskal-Wallis test with Dunn’s method; n = 8–10 (CD86^−/−^ NOD), n = 9 (NOR/LtJ), n = 10 (C57Bl/6). * p ≤ 0.05, ns: not significant.

We first conducted a pilot experiment, where we observed the time course of SAPP in CD86^−/−^ NOD mice in order to replicate the results of Salomon *et al*.^18^. We compared behavioral and electrophysiological measurements from CD86^−/−^ NOD mice to two control strains (NOR/LtJ and C57Bl/6 N). One NOR/LtJ animal had to sacrificed upon arrival due to a macroscopic tumor. CD86^−/−^ NOD mice showed a slower weight gain compared to the control animals and lost weight with the beginning of clinical symptoms after 23 weeks of age compared to control mice (2-way ANOVA, F_(12,173)_ = 8.046, p = 0.001; Fig. 1A). CD86^−/−^ NOD mice started showing clinical symptoms of paralysis as early as 25 weeks of age (Fig. 1B) with clinical scores gradually increasing (Fig. 1C). Two CD86^−/−^ NOD mice died due to unknown causes over the course of the experiment at 23 and 28 weeks of age, respectively (data not shown). None of the animals showed signs of mechanical hypersensitivity (Fig. 1D), but locomotor function significantly decreased in CD86^−/−^ NOD mice (Kruskal-Wallis test, p = 0.0018 (early) and p < 0.0001 (late); Fig. 1E). In parallel, CD86^−/−^ NOD mice showed a significant increase in F-wave latency of the sciatic nerve by 40% ± 7% (2-way ANOVA, F_(2,72)_ = 8.632, p < 0.0001; Fig. 1F) and a decrease of the sensory conduction velocity (SCV) and sensory nerve action potential (SNAP) amplitude (both Kruskal-Wallis test, p < 0.0001 (SCV) and p < 0.0239 (SNAP); Fig. 1G,H). Our data replicate and extend the results of Salomon *et al*. with mice exhibiting classic CIDP-like clinical, functional and electrophysiological features.

### Influence of fingolimod treatment on disease progression and locomotor function in CD86^−/−^ NOD mice

**Figure 2:**
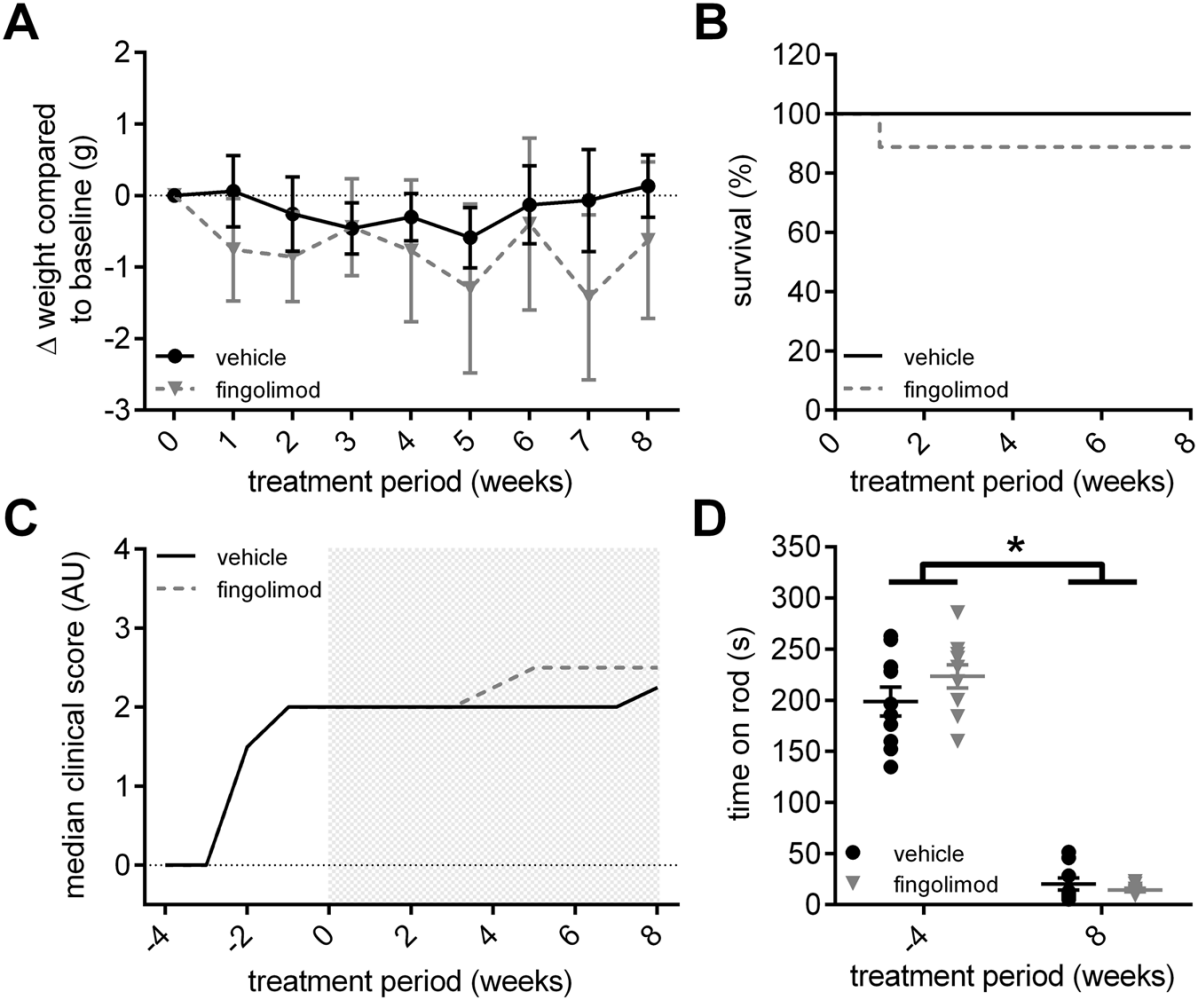
Effect of fingolimod treatment on disease progression. (**A**) Fingolimod (1 mg/kg bodyweight/day) and vehicle treated CD86^−/−^ NOD knockout mice showed no difference in weight progression over the course of the treatment. (**B**) Survival of mice did not differ between fingolimod and vehicle treated animals, although one animal in the fingolimod group died due to complications of the pump implantation. (**C**) Mice in both groups had a median clinical score of 2.0 at the start of treatment. Both fingolimod and vehicle treated mice showed a similar disease progression over the course of the 8-week treatment (indicated by gray shaded rectangle). (**D**) Locomotor function declined similarly between fingolimod and vehicle treated mice as disease and paralysis progressed. Statistical analysis: (**A**) 2-way ANOVA with Sidak post hoc test, (**B**) Kaplan-Meier analysis with Mantel-Cox test, (**D**) Kruskall-Wallis test with Dunn’s method; group sizes: n = 10 (vehicle), n = 9–10 (fingolimod). *p ≤ 0.05, ns: not significant.

After establishing the CD86^−/−^ NOD model in our lab as well as reliable functional and electrophysiological parameters to determine disease progression and severity in these mice, we tested the influence of the sphingosine-1-phosphate analogue fingolimod in this transgenic model. Fingolimod binds to the sphingosine-1-phosphate (S1P-)-receptors S1P1 > S1P3, S1P4 and S1P5 on T- and B-lymphocytes. Upon binding S1P1 becomes internalized, which sequesters lymphocytes in lymph nodes, preventing them from infiltrating peripheral organs and/or the nervous system. Fingolimod is currently used for treatment of multiple sclerosis. However, it was previously shown that prophylactic fingolimod treatment also ameliorates disease course and severity in an animal model of Guillain-Barré-Syndrome, experimental autoimmune neuropathy (EAN)^19^. To closely simulate clinical practice, we randomized CD86^−/−^ NOD mice to either fingolimod or vehicle treatment when animals reached a clinical score of ≥1.5, corresponding to unilateral hind limb weakness. Fingolimod was delivered continuously via an intraperitoneal osmotic pump at 1 mg/kg bodyweight/day and treatment was carried out for a total of eight weeks. Both groups showed a slight weight loss over the course of the treatment, which was not significant between the treatment groups (Fig. 2A). Survival rate was also not significantly different between fingolimod and vehicle treatment, although one animal in the fingolimod group died due to complications during pump implantation (Fig. 2B). Median clinical score at start of the treatment was 2.0 for both groups. Mice of either group showed a similar disease progression over the course of the treatment with a moderate increase of the clinical score and this was not different between groups (Fig. 2C). Due to increasing paralysis, locomotor function severely declined in both groups over the course of the treatment (Fig. 2D).

### Electrophysiological characteristics of CD86^−/−^ NOD mice undergoing fingolimod treatment

**Figure 3:**
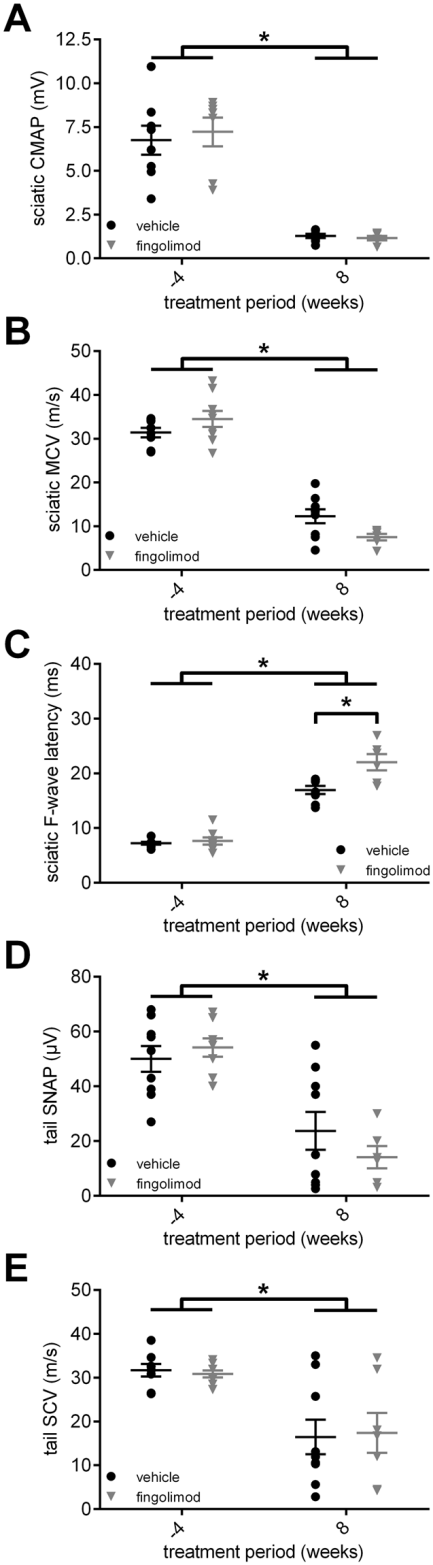
Electrophysiological characteristics of fingolimod treatment in CD86^−/−^ NOD mice. (**A**) The compound motor action potential amplitude (CMAP) of the sciatic nerve declined similarly in both fingolimod and vehicle treated CD86^−/−^ NOD mice. (**B**) Motor fibers showed electrophysiological signs of demyelination indicated by the decrease of motor nerve conduction velocity (MCV), which was more pronounced in the fingolimod treated mice compared to vehicle treatment. (**C**) F-wave latency increased in both groups with disease progression. Again, fingolimod treated mice were more affected than mice in the vehicle group. Similar to motor fibers, the (**D**) sensory nerve action potential amplitude (SNAP) and (**E**) sensory nerve conduction velocity (SCV) declined in both treatment groups. Statistical analysis: (**A**) Kruskall-Wallis test with Dunn’s method, (**B**–**E**) 2-way ANOVA with Sidak post hoc, group sizes: n = 10 (vehicle), n = 9–10 (fingolimod). *p ≤ 0.05, ns: not significant.

Demyelination of peripheral nerves was monitored *in vivo* with repeated electrophysiological measurements. In both groups, the amplitude of the compound motor action potential (CMAP) of the sciatic nerve showed a similar decline compared to baseline values before the start of treatment (Kruskal-Wallis test, p = 0.0105 (vehicle) and p = 0.0019 (fingolimod); Fig. 3A). The motor nerve conduction velocity (MCV) of the sciatic nerve also decreased in both groups (2-way ANOVA, F_(1,28)_ = 233.8, p < 0.0001); Fig. 3B). At the end of the treatment period, we observed a decline of the MCV of −78% ± 5% in the fingolimod group vs. −62% ± 15% in the vehicle group (unpaired t-test, p = 0.0248; data not shown). In parallel, the F-wave latency of the sciatic nerve increased significantly with disease progression. Interestingly, fingolimod treated mice also showed a prolonged F-wave latency at the end of the treatment period compared to vehicle treated mice (2-way ANOVA, F_(1,26)_ = 8.352, p = 0.0013; Fig. 3C). When we analyzed the sensory fibers, we observed a decline of the tail nerve sensory nerve action potential amplitude (SNAP) as well as the sensory nerve conduction velocity (SCV) compared to baseline in both groups without any differences between fingolimod or vehicle treatment (both 2-way ANOVA, SNAP: F_(1,30)_ = 28.48, p = 0.0092 (vehicle) and p = 0.002 (fingolimod); SCV: F_(1,30)_ = 20.30, p = 0.0135 (vehicle) and p = 0.0285 (fingolimod); Fig. 3D,E). In summary, CD86^−/−^ NOD mice developed a sensory-motor axonal-demyelinating neuropathy as described previously. However, we observed additional negative effects of fingolimod therapy on myelination as indicated by the differences in MCV and F-wave latency between fingolimod and vehicle treated mice.

### Influence of fingolimod on serum cytokine expression

**Figure 4:**
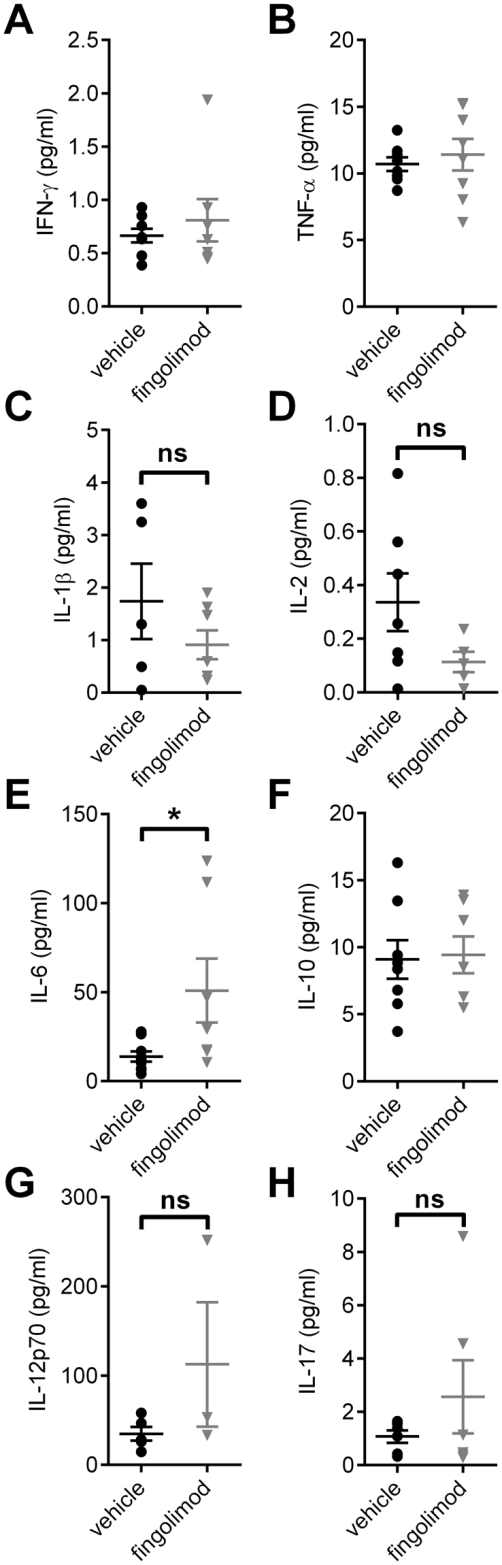
Influence of fingolimod on serum cytokine expression. Serum samples were taken after an eight-week treatment period with fingolimod or vehicle. No differences in cytokine levels were observed for (**A**) Interferon-γ (IFN-γ), (**B**) Tumor necrosis factor-α (TNF-α), (**C**) Interleukin (IL)-1b (IL-1b), (**D**) IL-2. (**E**) Fingolimod treated mice showed elevated levels of IL-6 compared to vehicle treated control animals, while (**F**) IL-10, (**G**) IL-12p70 and (**H**) IL-17 were not different between treatment groups. Statistical analysis: (**A**,**C**,**D**,**E**,**G**,**H**) Mann-Whitney-U test, (**B**,**F**) unpaired t-test, group sizes: n = 5–9 (vehicle), n = 3–8 (fingolimod). *p ≤ 0.05, ns: not significant.

Last, we investigated cytokine levels in serum samples taken at the end of the treatment period. Fingolimod treated and control mice showed no differences in the quantitative levels of Interferon-γ (IFN-γ), Tumor necrosis factor-α (TNF-α), Interleukin (IL)-1, IL-2, IL-10, IL-12p70 and IL-17 (Fig. 4A–D and F–H). However, there was a significant increase in the IL-6 expression in the fingolimod treated mice compared to control (Mann-Whitney-U test, p = 0.0229; Fig. 4E).

In conclusion, our data demonstrate that therapeutic fingolimod treatment does not improve clinical disease progression but rather worsens demyelination as assessed by electrophysiological parameters in this transgenic mouse model of CIDP.

## Discussion

In this study, we describe functional and electrophysiological characteristics of a CD86 knockout in NOD mice which closely mimic clinical findings of CIDP. We were able to replicate the data from Salomon *et al*. showing that a homogenous knockout of CD86 in NOD mice leads to distinct functional and electrophysiological characteristics that show great similarities to CIDP^18^. We expand the data by demonstrating that more sensitive electrophysiological parameters of demyelination such as F-wave latency and motor conduction velocity are severely affected in this model, which is also the case in CIDP patients. While many of the SAPP symptoms can also be observed in CIDP patients, disease progression is continuous and slow in this transgenic model, which can result in practical limitations when evaluating therapeutic options. Other animal models of spontaneous autoimmune neuropathy face similar difficulties^13^.

We used the CD86 knockout model to evaluate the therapeutic potential of the sphingosine-1-phosphate analogue fingolimod. Continuous intraperitoneal fingolimod treatment had no influence on disease progression or locomotor function. At first glance our data appears to contradict a study by Kim *et al*., who reported a positive effect of a four-week oral fingolimod therapy in the same transgenic mouse model^20^. However, in the study by Kim *et al*., fingolimod treatment began at seven months of age, independent of the individual clinical score, when most of the animals in the fingolimod group exhibited tail weakness and only few animals showed motor deficits in the hind limbs. In contrast, in the present study treatment began when mice showed signs of motor impairment in at least one of the hind limbs, a time point which was chosen to closely mimic clinical treatment conditions of CIDP patients. One explanation for the contradictory results therefore could be that demyelination had most likely further progressed in our study compared to the experiment of Kim *et al*. It was previously shown that fingolimod treatment can result in a quantitative reduction of myelin formation and is associated with apoptosis of Schwann cells^21^. Additionally, fingolimod seems to decrease immunoregulation by M2 macrophages, which are beneficial for tissue repair^22^. Thus, fingolimod might interfere with *remyelination* after peripheral nerve injury. We can in part substantiate this hypothesis by showing that MCV and F-wave latency were negatively affected in mice after fingolimod treatment compared to vehicle treatment.

CD4+ FoxP3+ regulatory T cells are also of great importance in the pathogenesis of autoimmune neuropathies as depletion of these cells leads to a more severe phenotype and enhanced CD3+ T cell infiltration of peripheral nerves^23^. The positive influence of CD4+ FoxP3+ regulatory T cells on *remyelination* was proven in demyelinating central nervous system disorders such as multiple sclerosis^24,25^. While it was shown in patients with multiple sclerosis that fingolimod decreases the amount of circulating CD4+ T cells, the remaining CD4+ T cells were enriched with regulatory FoxP3+ cells^26^. This could explain why *prophylactic* fingolimod treatment was beneficial in rodent models of autoimmune neuritis^16^. However, the increase of regulatory T cells that possibly occurs during fingolimod treatment might not be sufficient to outweigh its impact on repair mechanisms at a later time point of the disease.

In summary, we argue that fingolimod may have beneficial effects in autoimmune neuropathies, but only at very early stages of the disease, which leaves a narrow therapeutic time window in this condition. This would explain the recently obtained results of a phase II/III double-blind randomized clinical trial with 106 CIDP patients: Although detailed results have not been published yet, investigators have disclosed that the time CIDP patients spent on the fingolimod or placebo medication until clinical progression (increase of INCAT overall disability score, primary endpoint) did not differ between the treatment groups^27^. In addition, there was a significant amount of patients who stopped the trial prematurely due to side effects^28^. As CIDP diagnosis is often delayed and patients have progressed to paresis at the point of treatment initiation, it might be a possible explanation why fingolimod was not superior to placebo in this clinical trial. However, a more detailed subanalysis of the trial will demonstrate whether fingolimod can influence secondary outcome parameters and whether a subset of patients benefitted from the fingolimod medication.

## Materials and Methods

### *In vivo*

#### Animal numbers, housing conditions and surveillance of clinical symptoms

We chose a previously described transgenic mouse model with knockout of CD86 (CD86^−/−^) in mice of the non-obese diabetic (NOD) background which leads to the spontaneous development of an autoimmune peripheral polyneuropathy (SAPP) and mimics clinical and histological characteristics of chronic inflammatory demyelinating polyradiculoneuropathy (CIDP)^18^. A total of 30 14-week old female CD86^−/−^ NOD mice (NoD.129S4-Cd86^*tm1Shr*^/JbsJ; purchased from Jackson Laboratory, Bar Harbor, ME, USA and bred at Charité Universitätsmedizin Berlin, Research Department of Experimental Medicine (FEM), Berlin, Germany), 10 13-week old female NOR/LtJ and 10 10-week old female C57BL/6 N mice from Charles River (Sulzfeld, Germany) were used for this study. Successful knockout of CD86 was confirmed via polymerase-chain reaction (PCR) of tail biopsies prior to experiments. All experimental procedures conformed to animal welfare guidelines and were previously approved by an official committee (Landesamt für Gesundheit und Soziales, Berlin, Germany). Mice were housed in groups of five in an enriched environment and allowed unlimited access to food and water. Animals were maintained on a 12:12 hour light/dark cycle and behavioral testing was conducted between 10 am and 4 pm. The general wellbeing of the mice as well as their weight and disease progression were assessed daily and clinical symptoms rated using an arbitrary score ranging from 0 to 5 corresponding to the following definitions: 0 = healthy, 0.5 = light tail weakness, 1.0 = tail paralysis, 1.5 = unilateral hind limb weakness, 2.0 = bilateral hind limb weakness, 2.5 = bilateral hind limb paralysis, 3.0 = unilateral hind limb plegia or fore limb weakness, 3.5 = bilateral hind limb plegia, 4.0 = hind limb plegia and bilateral fore limb paralysis, 4.5 = bilateral hind limb plegia and unilateral plegia of fore limbs, 5.0 = tetraplegia or death due to neurological symptoms. In accordance with animal welfare guidelines, animals were sacrificed when reaching a clinical score of > 3.5.

#### Sample sizes and methods of randomization and blinding

Two different sets of animal experiments were performed: (1) In the initial trial, we monitored spontaneous disease progression in 10 female 14-week old CD86^−/−^ NOD mice. Wildtype NOD mice develop type-1 diabetes^29^ and due to the likelihood of a diabetic polyneuropathy in these animals, we used 10 female NOR/LtJ and 10 female C57Bl/6 N mice as control animals to compare clinical, behavioral and electrophysiological symptoms of CIDP in CD86^−/−^ NOD mice. Samples sizes were determined with G*Power 3 statistical software^30^ according to the published effects sizes with an α-error of 0.05 and a desired power of 0.8. (2) In the second trial, onset of clinical symptoms and disease progression was monitored in 20 female 14-week old CD86^−/−^ NOD mice. When reaching a clinical score of ≥1.5 ( = unilateral hind limb weakness), mice were randomly assigned to two different groups and treated with fingolimod (1 mg/kg bodyweight/day) or vehicle for eight weeks. We determined the group size based on the effects sizes of electrophysiological measurements in the pilot experiment with a desired power of 0.8 and an α-level of 0.05 using G*Power 3 statistical software. The investigators conducting the behavior and electrophysiological experiments were blinded throughout the entire process including statistical analysis.

#### Treatment protocols, drug application and placement of osmotic pump

Fingolimod (S5002; Selleckchem, Munich, Germany) was dissolved in 0.9% saline solution (NaCl) to a final concentration of 9.5 mg/ml. Reservoirs (100 µl) of osmotic pumps (model 1004; Alzet osmotic pumps, Cupertino, CA, USA) were filled either with fingolimod solution or vehicle (0.9% NaCl) according to the manufacturer’s instructions. Mice were briefly anaesthetized in isoflurane (1.3–1.5% (vol/vol) and 50% O_2_). A small incision was made in the abdominal skin and peritoneum and the pump placed inside. The peritoneum was closed using absorbable sutures. After four weeks, the pumps were exchanged resulting in a continuous fingolimod or vehicle treatment at 1 mg/kg bodyweight/day over the course of eight weeks.

#### Behavior analysis

Upon arrival in the animal facility, animals were allowed to acclimate for seven days and thereafter handled for five consecutive days to familiarize the mice to the investigators. Cages and animals were randomly selected for behavioral testing and all behavior tests were carried out in a dedicated laboratory with soundproof chambers. The RotaRod test was used to determined locomotor ability and carried out as previously described^31^. Briefly, mice were put on a rotating rod which gradually increases speed from 4 to 40 rpm in 300 s and the latency to fall off the rod was automatically measured by a floor sensor (TSE Systems GmbH, Bad Homburg, Germany). Baseline recording was obtained after a four-day training period in the task. At every time point, three trials per animal were averaged.

The mechanical withdrawal threshold was measured as described previously^31,32^ using a hand-held force transducer fitted with a 0.5 mm^2^ polypropylene probe (IITC, Woodland Hills, CA, USA). Investigators were trained to apply semi-flexible filaments to the center of each hind paw with gradually increasing pressure until a clear withdrawal response was visible. If an animal did not show a clear withdrawal response within 5 s the trial was repeated. The force used that elicited the withdrawal response (mechanical withdrawal threshold in grams (g)) was automatically recorded by the device. The maximally applied force was 10 g. For each time point, three trials of each paw were averaged.

#### Electrophysiological measurements

Sensory nerve conduction velocity (SCV) and sensory nerve action potential amplitudes (SNAP) of the tail nerve were measured in ketamine/xylazine anaesthesia with a customized Neurosoft Evidence 3102evo EMG/ENG device (Schreiber & Tholen Medizintechnik GmbH, Stade, Germany). Stimulation needle electrodes were applied at the base of the tail with the recording electrodes five cm distal. 50 stimuli of 0.1 ms each were applied at supramaximal stimulation intensity and averaged to obtain SNAP and SCV^33^. Sciatic nerve compound motor action potential (CMAP) amplitudes and motor nerve conduction velocity (MCV) as well as F-waves were measured according to a previously described protocol in ketamine/xylazine anesthesia^34^: The tibial and peroneal nerve were stimulated with supramaximal intensity at the ankle (distal). Afterwards the sciatic nerve was stimulated proximal at the sciatic notch. CMAPs were recorded with steel needle electrodes in the foot muscles. MCV was calculated by measuring the distance between the distal and proximal stimulation electrodes. Afterwards, 10 repetitive supramaximal stimuli were applied at the proximal stimulation electrodes (sciatic notch) and recorded from the foot muscles to obtain F-wave latencies and persistence. For statistical analysis, the shortest F-wave latency from each mouse was used to limit experimental bias.

### Cytokine profiling

Whole blood samples were kept upright for 1 h on ice and thereafter centrifuged at 3,000 g for 10 min. Serum was collected and cytokines analyzed using customized multiplex ELISA plates (Meso Scale Diagnostics, Rockville, MD, USA) according to the manufacturer’s protocol.

### Data processing, data availability, exclusion criteria and statistical analysis

The manuscript was prepared in accordance with ARRIVE guidelines^35^. Data is presented as mean ± sem in aligned dot plots. Clinical scores are shown as median. Data processing and analysis were completed before unblinding of the analyzer. Gaussian distribution of data was checked prior to statistical analysis using Shapiro-Wilk normality test. Statistical analysis was performed using Prism v6.0 (GraphPad Software, La Jolla, CA, USA). Normally distributed data was analyzed using unpaired two-sided t-tests (2 groups) or 2-way ANOVA with Sidak post hoc test (≥2 groups over time). Not normally distributed data was analyzed with Mann-Whitney-U Test (2 groups) or Kruskal-Wallis test with Dunn’s method (≥2 groups over time). p < 0.05 was considered statistically significant and is depicted by an asterisk (ns: not significant). Data was checked prior to statistical analysis for outliers using Peirce’s criterion^36,37^. The dataset generated and analyzed during the current study are available from the corresponding author on reasonable request.
